# Soluble syndecan-3 binds chemokines, reduces leukocyte migration in vitro and ameliorates disease severity in models of rheumatoid arthritis

**DOI:** 10.1186/s13075-019-1939-2

**Published:** 2019-07-12

**Authors:** Andrew D. Eustace, Emily F. McNaughton, Sophie King, Oksana Kehoe, Andreas Kungl, Derek Mattey, Angela H. Nobbs, Neil Williams, Jim Middleton

**Affiliations:** 1 Bristol Dental School, University of Bristol, Lower Maudlin Street, BS1 2LY Bristol, UK; 2 Leopold Muller Arthritis Research Centre, Medical School, RJAH Orthopaedic Hospital, ISTM, Keele University, Oswestry, UK; 3 Institute of Pharmaceutical Sciences, Karl-Franzens-University Graz, Humboldtstrasse 46, A-8010 Graz, Austria; 4 Staffordshire Rheumatology Centre, Haywood Hospital, Stoke-on-Trent, UK; 5 School of Cellular and Molecular Medicine, University of Bristol, Biomedical Sciences Building, BS8 1TD Bristol, UK

**Keywords:** Syndecan-3, Cell migration, Therapeutic, Chemokines, Animal model

## Abstract

**Background:**

Syndecans are heparan sulfate proteoglycans that occur in membrane-bound or soluble forms. Syndecan-3, the least well-characterised of the syndecan family, is highly expressed on synovial endothelial cells in rheumatoid arthritis patients. Here, it binds pro-inflammatory chemokines with evidence for a role in chemokine presentation and leukocyte trafficking into the joint, promoting the inflammatory response. In this study, we explored the role of soluble syndecan-3 as a binder of chemokines and as an anti-inflammatory and therapeutic molecule.

**Methods:**

A human monocytic cell line and CD14+ PBMCs were utilised in both Boyden chamber and trans-endothelial migration assays. Soluble syndecan-3 was tested in antigen-induced and collagen-induced in vivo arthritis models in mice. ELISA and isothermal fluorescence titration assays assessed the binding affinities. Syndecan-3 expression was identified by flow cytometry and PCR, and levels of shedding by ELISA.

**Results:**

Using in vitro and in vivo models, soluble syndecan-3 inhibited leukocyte migration in vitro in response to CCL7 and its administration in murine models of rheumatoid arthritis reduced histological disease severity. Using isothermal fluorescence titration, the binding affinity of soluble syndecan-3 to inflammatory chemokines CCL2, CCL7 and CXCL8 was determined, revealing little difference, with K_d_s in the low nM range. TNFα increased cell surface expression and shedding of syndecan-3 from cultured human endothelial cells. Furthermore, soluble syndecan-3 occurred naturally in the sera of patients with rheumatoid arthritis and periodontitis, and its levels correlated with syndecan-1.

**Conclusions:**

This study shows that the addition of soluble syndecan-3 may represent an alternative therapeutic approach in inflammatory disease.

**Electronic supplementary material:**

The online version of this article (10.1186/s13075-019-1939-2) contains supplementary material, which is available to authorized users.

## Background

Syndecans are heparan sulfate proteoglycans (HSPG) that consist of a core protein with heparan sulfate (HS) glycosaminoglycan (GAG) chains covalently attached. They form part of the glycocalyx network of membrane-bound proteoglycans and glycoproteins at the endothelial surface, coating the luminal surface of the blood vessels [1]. There are four members of the syndecan family [1–4] all of which contain three domains: an extracellular, transmembrane and cytoplasmic domain. Extracellular variations exist in the length of the core protein, as well as the type and number of glycosaminoglycan chains attached [2]. The intracellular components contain conserved regions shared by all syndecan family members but also a variable region thought to interact with a range of binding partners including kinases, GTPases and cytoskeletal molecules that may be syndecan or tissue specific [3, 4].

In the process of inflammation, HSPGs bound to endothelial cell (EC) membranes have been shown to play pro-inflammatory roles [5–7]. In the leukocyte transmigration cascade, HSPGs stabilise rolling through interacting with l-selectin, present chemokines on the endothelial surface (resulting in leukocyte integrin activation) and are also involved in facilitating chemokine transcytosis to the luminal endothelial surface [8–13]. There are, however, a number of contrasting studies using syndecan knockout mice (syndecan-1, syndecan-3 and syndecan-4) in various disease models that also suggest an anti-inflammatory syndecan function [14–16]. A study by Kehoe et al. identified a dual functionality of syndecan-3 being pro-inflammatory in the joint and anti-inflammatory in the skin contributing to the idea that the syndecan role may be tissue or inflammatory state specific [16].

Syndecans are also known to be shed from the endothelial surface in a range of chronic inflammatory conditions. It is proposed that shedding can serve a range of functions including the removal of cells from points of focal adhesion to the extracellular matrix, removing ligands associated with endothelial HS which could create paracrine effectors, or to release soluble syndecans that can compete for ligands with their membrane-bound counterparts [17–20]. While the true function of shedding may not yet fully be understood, increases in serum concentrations of syndecan-1 have been detected in chronic kidney disease [21], inflammatory bowel disease [22], systemic lupus erythematosus [23] and periodontitis [24]. As these diseases all involve damage to endothelial surfaces, an increase in the serum levels could simply represent and correlate with a disruption or destruction of the glycocalyx. One such inflammatory disease is rheumatoid arthritis (RA), a chronic, inflammatory, systemic autoimmune disease affecting approximately 1% of the general population. Inflammation is central to the RA pathology which can cause significant disability and mortality, not just through joint disease manifestation, but also through an increased association with cardiovascular disease. The hallmark feature of RA is chronic inflammation of the joint synovium, characterised by mass infiltration of activated leukocytes that contribute to progressive destruction of cartilage and bone. Chemokines play a role facilitating this increase in infiltration, and as such, their expression levels are naturally found to be elevated in sera, synovial fluid and the synovium in human RA [25, 26]. A variety of CC and CXC chemokines are produced by synovial macrophages and other cell types that attract neutrophils and subsequently more long-lived leukocytes. These chemokines include CCL2, CCL3, CCL5, CCL7, CCL8, CCL13, CCL14, CCL15, CCL16, CXCL1 and CXCL8 [27]. While the therapeutic landscape regarding RA treatment has been positively transformed in the past few decades through combination therapies of disease-modifying anti-rheumatic drugs and biologic response modifiers, only 50–60% of RA patients report long-standing benefits. This figure highlights the complexity of RA but also a gap in our understanding of the disease and the mechanism required to develop more effective treatments.

In an attempt to identify which syndecans are responsible for chemokine binding in chronic inflammatory diseases, Patterson et al. characterised the changes in HSPG expression in human RA synovium [28]. They found that not only was there an increase in syndecan-3 expression, but also that there was a selective induction of a CXCL8 chemokine-binding site on this HSPG on ECs not present in healthy controls [29]. Furthermore, in an RA mouse model, syndecan-3 increased leukocyte accumulation and disease severity [16]. These studies suggest that membrane-bound endothelial syndecan-3 presents chemokines to the blood leukocyte resulting in leukocyte migration into the RA synovium. However, little is known about the role of soluble syndecans in inflammation. That is whether they bind chemokines, alter chemokine-driven leukocyte migration and if they could act as competitors and therapeutic agents. The least characterised in the syndecan family is syndecan-3, first identified as the predominant syndecan of the nervous system [30]. It is also known to contribute to cartilage and skeletal muscle cell differentiation [31] as well as being implicated in the control of feeding behaviour [32].

The research presented here characterises the role that soluble syndecan-3 plays in inflammation through analysing chemokine binding, mRNA and protein expression; exploring shedding patterns; and in addition, evaluating the therapeutic potential of soluble syndecan-3 in vitro and in vivo.

## Methods

### ELISA

Heparin/GAG binding ELISA plates (Iduron, Manchester, UK) were used to determine the difference in chemokine binding to syndecan-3 (R&D Systems, Abingdon, UK) and HS (Sigma-Aldrich, Dorset, UK). The ELISA was carried out according to the manufacturer’s instructions. Briefly, 200 μl of HS (25 μg/ml; Sigma-Aldrich) or syndecan-3 (25 μg/ml; R&D Systems) diluted in standard assay buffer (SAB; 100 mM, 50 mM sodium acetate, 0.2% *v*/*v* Tween 20) were added to wells in the GAG binding microplate overnight. Plates were washed with buffer and blocked with blocking solution (PBS/1% bovine serum albumin, BSA). A dilution series for the chemokine CCL7 (from 0 to 3 μg) was generated, and 200 μl was added to the wells for 2 h. Plates were washed with buffer and incubated with 200 μl of biotinylated anti-CCL7 (250 ng/ml; Peprotech) for 1 h. Plates were washed again and incubated with 200 μl of ExtrAvidin-alkaline phosphatase (4 μg/ml; Sigma-Aldrich) for 30 min. They were washed a final time, and development reagent was added (SigmaFAST p-Nitrophenyl phosphate) for 40 min before optical densities were read at 405 nm on a microplate reader. OD_405_ readings from a blank well with no syndecan-3 or HS were subtracted from each test sample to correct for background OD interference. OD was plotted against CCL7 concentration to give two standard curves.

In a separate assay, TNFα-supplemented medium used to stimulate hBMECs was also tested for human syndecan-3 by ELISA (R&D Systems) in accordance with the manufacturer’s protocol. Control medium with no TNFα (DMEM-F12/0.5% FBS) and TNFα-supplemented medium [100 ng/ml] were incubated with hBMECs for 1, 2, 6 and 24 h.

### Isothermal fluorescence titration

Isothermal fluorescence titration (IFT) was used to study the binding affinity of syndecan-3 and HS to CCL2, CCL7 and CXCL8. The technological principle is based on the reduction in fluorescence (quenching) of intrinsic tryptophan amino acids within each chemokine when syndecan-3 binds as described elsewhere [33]. Titration experiments were performed on a Fluoromax-4 Spectrofluorometer (Horiba, Kyoto, Japan). Protein fluorescence emission spectra were recorded over the range of 300–400 nm with excitation at 295 nm. Slit widths were set at 4 nm + 4 nm for excitation and emission. The use of concentrated GAG oligosaccharide/syndecan-3 stock solution ensured a dilution of the protein sample less than 5%. Chemokine (CCL2, CCL7 and CXCL8) solutions (700 nM) were prepared from stock solutions and needed to be equilibrated for 30 min in PBS. Respective ligands (syndecan-3, HS) were added in 20 nM concentrations until maximum quenching was reached corresponding to saturation of all binding sites. The chemokine solutions were equilibrated for 1 min, and fluorescence emission spectra were collected. For background correction, the emission spectra of the respective ligand concentrations were collected in PBS buffer only. They were subsequently subtracted from the protein emission spectra and divided by the value given at the added HS concentration. Values from this equation were averaged from three independent experiments and plotted against HS concentration [33]. The resulting binding isotherms were analysed by nonlinear regression to give a K_d_ using the program Origin (Microcal Inc., MA, USA).

### Cell culture

For in vitro migration assays and mRNA and protein expression analysis, immortalised human bone marrow endothelial cells (hBMEC) donated by Prof. BB Weksler [34] were utilised. These cells express relevant adhesion molecules and support leukocyte trans-endothelial migration [35]. Cells were maintained in DMEM-F12 (Lonza Ltd., Slough, UK) containing 10% FBS (Sigma-Aldrich) at 37 °C in 5% CO_2_ atmosphere and grown to around 70% confluence before being used in experiments. The human monocytic cell line (THP-1) was grown in RPMI-1640 (Sigma-Aldrich) supplemented with 10% FBS (Sigma-Aldrich). Cells were split at a 3:1 ratio once they had exceeded a density of 1 × 10^6^ cells/ml. PBMCs were isolated from healthy volunteers via Polymorphprep separation technique (Axis-Shield Diagnostics Ltd., Dundee, UK).

### Chemotaxis

Two different chemotaxis systems were utilised, both operating with a two-chamber system: 48-well micro chemotaxis Boyden chamber (Neuro Probe, Inc., MD, USA) and the 96-transwell system (Corning Ltd., Flintshire, UK) with the latter containing a hBMEC monolayer between the chambers.

#### Boyden chamber chemotaxis

RPMI (100 μl) supplemented with 1% BSA was added to both upper and lower chambers of the Boyden chamber and allowed to block for 30 min before being removed. Twenty-eight microlitre of CCL7 (100 ng/ml; optimum concentration was ascertained from adding 0.01–500 ng/ml CCL7 in separate Boyden chamber experiments; data not shown) or RPMI alone (negative control) was added in triplicate to the basal chambers with an 8-μm polycarbonate filter applied on top. The upper chamber was then attached. THP-1 cells (50 μl) at a concentration of 1.6 × 10^6^ cells/ml were added to the upper chamber for an incubation time of 3 h at 37 °C and 5% CO_2_ atmosphere.

After 3 h, the suspensions in the upper chambers were removed and the top of the filter was wiped gently. Cells/media in the bottom chamber were then counted using a haemocytometer (Immune systems Ltd., Devon, UK). Migrated cells were identified as those within the basal chamber media, and migration was calculated as a percentage of the positive control. The method used here is a modified version of a previously described assay [36].

Further experiments saw the addition of syndecan-1, syndecan-2, syndecan-3 or syndecan-4 (R&D Systems) to the lower chambers with the chemokine to determine any pro- or anti-migratory effects. Syndecan concentrations ranged from 0.01 to 100 μg/ml.

#### Endothelial transmigration

3.2 × 10^4^ ECs were seeded onto 96-well chamber membranes (Corning Ltd.) in DMEM-F12/10% FBS. The confluence of the monolayers was tested via trans-endothelial electrical resistance (TEER). After 24 h (at peak resistance), adhered cells were washed gently with warm PBS twice. Two hundred thirty-five microlitre of CCL7 (100 ng/ml) was added to the basal chamber. The apical chamber was filled with 1 × 10^5^ human PBMCs in 100 μl DMEM-F12/0.5% FBS and allowed to migrate for 3 h at 37 °C CO_2_ atmosphere. Migrated cells were classified as those which had migrated to the bottom chamber. These cells were then diluted with trypan blue (Sigma-Aldrich) and counted using a haemocytometer. Once again, further experiments saw the addition of syndecan-3 (0.1–50 μg/ml; R&D Systems), syndecan-3 core protein (a gift from Dr. James Whiteford, University of Queen Mary, University of London, UK) and HS chains (Sigma) to the lower chambers with CCL7 to determine their effects.

### Animals

Antigen-induced arthritis (AIA) experiments were undertaken on 8-week-old inbred male C57BL/6JOlaHsd wild-type mice (Harlan Laboratories Ltd., Bicester, UK). Procedures were conducted in accordance with Home Office Project Licence PPL40/3594 in collaboration with Personal Licence holder Dr. Oksana Kehoe at Liverpool John Moores University.

Collagen-induced arthritis (CIA) experiments were conducted on adult male DBA/1 wild-type mice (Charles River, Oxford, UK) in accordance with Home Office Project Licence PPL 30/3097 by KWS Biotest, Bristol, UK.

### Injection of syndecan-3 in mice

#### Antigen-induced arthritis

Murine AIA was induced as previously described [37]. Mice were immunised subcutaneously with 1 mg/ml of mBSA emulsified with an equal volume of Freund’s complete adjuvant (CFA) via intraperitoneal injection with 100 μl (160 ng) heat-inactivated *Bordetella pertussis* toxin in PBS. The immune response was boosted 1 week later with the same amount of mBSA as before. Twenty-one days after the initial immunisation, murine AIA was induced by intra-articular (IA) injection of 10 mg/ml mBSA in PBS in the right knee (test) joint. For a control, the same volume of PBS was injected into the left knee joint at the same time points.

Human syndecan-3 (5 μg; R&D Systems) in 10 μl PBS (500 μg/ml final concentration) was injected into the right knee joints at day 1 post-arthritis induction at peak joint swelling. A control group was injected with PBS alone at the same time point. Eight mice were randomly assigned to either the control (PBS injection) or test (syndecan-3 injection) group, and 4 from each group were sacrificed on day 3 and day 7 post-syndecan-3 injection. The dosing rationale was based on previous IA injections of a GAG-binding peptide that reduced joint swelling in the same mice (unpublished data).

#### Collagen-induced arthritis

CIA was induced as previously described [38]. On day 0, a subcutaneous injection was administered of 100 μl of an emulsion containing 100 μg of CII (type II collagen) in complete Freund’s adjuvant supplemented with 4 mg/ml *Mycobacterium tuberculosis* H37Ra. On day 21, animals received a subcutaneous injection with a booster emulsion containing 100 μg of CII in incomplete Freund’s adjuvant (IFA). Animals were treated according to the syndecan administration schedule below at days 21, 24, 29, 34, 39 and 44.

The results obtained from the AIA model were used as a starting point for intravenous (IV) injections in the CIA model. The effects observed in the AIA model came after a 5-μg injection into the IA space. The decision was made in CIA to administer 7 μg on 6 occasions every 3–4 days post-peak swelling, based on syndecan-3 being injected into the bloodstream as opposed to the smaller volume of the IA space. The administration volume for IV injection was 5 ml/kg. DBA/1 mice weighed approximately 20 g meaning an administration volume of 100 μl syndecan-3 in PBS (70 μg/ml final concentration; *n* = 10). Control animals (*n* = 10) were injected with 100 μl PBS. At termination (day 45), the hind limbs were dissected and stored in formaldehyde.

### Histological assessment

The knee joints (AIA) and hind paws (CIA) were removed and fixed in neutral buffered formal saline. Decalcification in formic acid, embedding in paraffin and sectioning were carried out by the histology department RJAH Orthopaedic Hospital (Oswestry, UK). Mid-sagittal serial sections (5 μm thickness) were cut and stained with haematoxylin and eosin (H&E) or toluidine blue. The sections were scored blind by two independent observers from 0 to 3 based on the severity of hyperplasia of the synovial lining layer, synovial exudate in the joint space and cartilage GAG depletion or 0–5 for synovial expansion as described in earlier AIA studies [16]. An additional parameter of pannus invasion being scored from 0 to 3 was used when assessing CIA. All scores were combined generating an arthritis index.

### Immunofluorescence

Unstained AIA knee sections were dewaxed in xylene twice for 5 min, rinsed in IMS twice for 2 min and rehydrated in distilled water for 5 min. Antigen retrieval was then carried out via immersion in 10 mM Tris buffer HCL (pH 9) overnight at 50 °C, and the slides were then washed in PBS.

The sections were stained with anti-CCL7 (R&D Systems; 10 μg/ml, goat anti-mouse, IgG) in PBS for 1 h. The slides were washed in PBS 3 times, and 200 μl of Alexa-594 donkey anti-goat IgG1 (1:200 dilution in PBS/10% mouse serum; Invitrogen) for a further 1 h. Cells were washed again 3 times in PBS and stained with 200 μl DAPI (2 μg/ml in PBS; Invitrogen) for 3 min before being washed in PBS for 5 min, rinsed in distilled water, air dried and mounted with Hydromount (Thermo Fischer Scientific).

### Flow cytometry

Flow cytometry was used to characterise syndecan-3 protein expression in response to TNFα. hBMECs were grown in chamber slides and stimulated with or without TNFα for 24 h (Peprotech EC Ltd., London, UK; 100 ng/ml in DMEM-F12, 10% FBS). Cells were harvested using cell dissociation buffer (Sigma-Aldrich) to ensure that extracellular proteins remained intact. To ascertain extracellular expression, one population of cells was blocked with FcR blocking agent in FACS buffer (Thermo Fisher Scientific, Loughborough, UK) and stained with anti-syndecan-3 (APC; 25 μg/ml; R&D Systems) or an isotype control (APC; 25 μg/ml; Thermo Fisher Scientific Ltd.) antibody and fixed according to the manufacturer’s protocol (FIX&PERM, Thermo Fisher Scientific). To determine intracellular expression, a duplicate population was blocked, stained with anti-syndecan-3, fixed, permeabilised and stained again according to the protocol. The level of expression from this population was subtracted from the non-permeabilised population. Both populations of cells were washed in FACS buffer, re-suspended in 1% PFA and stored at 4 °C prior to flow cytometry. Data are expressed as a median fluorescence intensity.

### mRNA expression of syndecan-3

To characterise syndecan-3 gene expression in response to an inflammatory stimulus, hBMECs were cultured in the presence of TNFα. hBMECs were grown in 8-well chamber slides (Thermo Fischer Scientific) in DMEM-F12/0.5% FBS alone or supplemented with TNFα [100 ng/ml] for 0, 1, 2, 6 and 24 h. RNA (~ 100 μg) was extracted from 3–4 × 10^5^ hBMECs using RNeasy mini kit (Qiagen, Hilden, Germany). Approximately 6–8 μg RNA was converted to cDNA using iScript cDNA synthesis kit (Biorad Laboratories Ltd., Hertfordshire, UK) according to the manufacturer’s instructions. Controls contained no RNA. The mRNA expression levels of relevant genes (syndecan-3 and glyceraldehyde 3-phosphate dehydrogenase; GAPDH) were quantified by real-time PCR using SYBR green supermix (Biorad) and read via a Chromo-4 Detector (Biorad) with primers as published elsewhere [29]. The sequences for syndecan-3 were ACCCCAACTCCAGAGACCTT (forward) and CCCACAGCTACCACCTCATT (reverse); the specific primers for GAPDH were GAGTCAACGGATTTGGTCGT (forward) and GACAAGCTTCCCGTTCTCAG (reverse). The total volume per well was 20 μl, containing 1–3 μg cDNA (100 fg–100 ng), 10 μl SYBR green and 0.8–1.16 μl (300 nM) forward and reverse primers (Eurofins Genomics, Ebersberg, Germany), with PCR water making up the final 20 μl volume as listed below.

Syndecan-3 mRNA expression was analysed in response to TNFα at time points 1, 2, 6 and 24 h. C_T_ values obtained for the reference gene GAPDH at each time point were subtracted from syndecan-3 gene expression to generate ΔC_T_. The ΔC_T_ control values (in the absence of TNFα) were then subtracted from TNFα-stimulated samples (ΔΔC_T_). These were then transformed to create 2^−ΔΔCT^. C_T_ values were subtracted from the reference gene (GAPDH) for at each time point. The resulting control values at each time point were then subtracted from the TNFα-stimulated values, of which a ratio was calculated via 2^−ΔΔCT^.

### Detection of syndecan-1 and syndecan-3 in human blood

RA patient serum collected from the Haywood Hospital in collaboration with Dr. Derek Mattey was analysed using syndecan-1 and syndecan-3 ELISA Duoset kits (R&D Systems) according to the manufacturer’s instructions. In addition, blood serum samples were taken from patients attending the Bristol Dental School clinic with a diagnosis of periodontitis.

### Statistics

All statistical analyses were performed using GraphPad Prism software (San Diego, CA). To determine statistical significance, Mann-Whitney *U* tests were performed on histological and ELISA data, excluding the ELISA data collected from culture media which was analysed via Student’s *t* test. The CCL7 ELISA was also analysed by *t* test and a two-way ANOVA with Sidak’s multiple comparisons post-test. Pearson’s correlation coefficient was used to analyse syndecan-1 and syndecan-3 correlation. One-way ANOVA with Tukey post-test C_T_ values were used when analysing mRNA data. For Boyden chamber and trans-endothelial migration results, an ANOVA with a Dunnet post-test was performed.

## Results

### Chemokines bind to soluble syndecan-3, comparison with HS

**Fig. 1:**
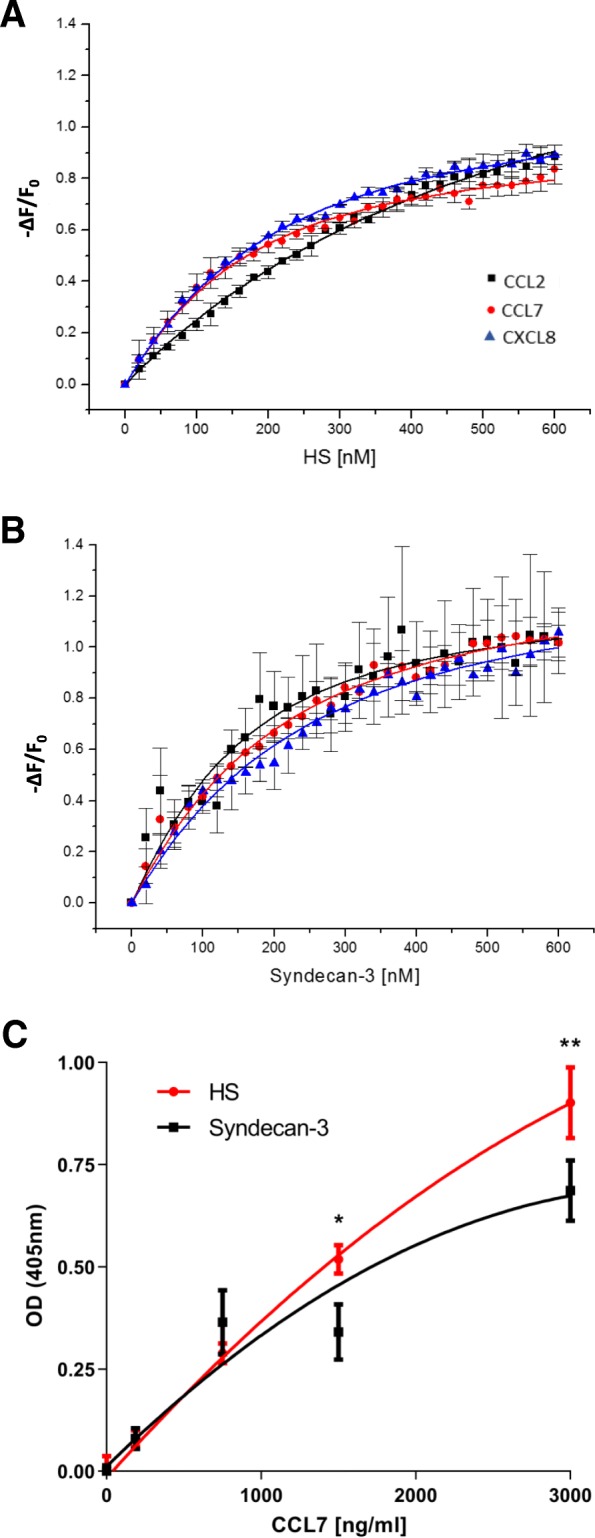
Chemokines bind to soluble syndecan-3 and heparan sulfate (HS). Isothermal fluorescence titration (A+B). **a** HS binding isotherms of CCL2 (black squares), CCL7 (red circles) and CXCL8 (blue triangles). **b** Syndecan-3 binding isotherms of the same chemokines. For K_d_ values, see Table 1. The *y*-axis shows the relative change in fluorescence intensity following ligand addition: Δ*F* = *F* (fluorescence emission at a certain ligand concentration) − *F*0 (fluorescence emission in the absence of ligand). **c** Syndecan-3 and HS were coated onto GAG microplates overnight [25 μg/m]. A dilution series of CCL7 was added to the plates to identify the level of binding. Plates were read at 405 nm. Data points represent means ± standard error (*n* = 3 separate experiments)

In an attempt to further the work carried out by Patterson et al. on syndecan-3 chemokine binding on ECs in the RA synovium [28, 29], we tested the binding affinities of clinically relevant chemokines to commercially sourced soluble syndecan-3 using IFT. Binding affinities (K_d_) between chemokines CCL2, CCL7and CXCL8 and syndecan-3 were in the low nM range and were similar (Fig. 1a, b; Table 1). Chemokine affinities varied more when binding to HS with the order being: CCL7>CXCL8>CCL2. A major difference was the reduced affinity of CCL2 for HS compared to syndecan-3 (over fivefold; Fig. 1a, b; Table 1). The results obtained from coating specialised ELISA plates with syndecan-3 and HS also showed chemokine binding (Fig. 1c), and taking the data overall, CCL7 binding was not significantly different between the two molecules. At lower and physiological CCL7 concentrations (< 1 μg/ml), the optical densities for syndecan-3 and HS were also not significantly different; however, at high concentrations (1.5 and 3 μg/ml), HS bound more chemokine (*P* < 0.05 and *P* < 0.01 respectively).

**Table 1:** Isothermal fluorescence values for CCL2, CCL7 and CXCL8 binding to HS and syndecan-3. Isothermal fluorescence values are expressed as dissociation constants (K_d_) with standard error of the mean

	Heparan Sulfate (HS)		Syndecan-3	
	K_d_ (nM)	± (SEM)	K_d_ (nM)	± (SEM)
CCL2	508	32	94	20
CCL7	109	8	143	14
CXCL8	145	6	184	22

### Soluble syndecan-3 decreases levels of THP-1 and PBMC migration in vitro

**Fig. 2:**
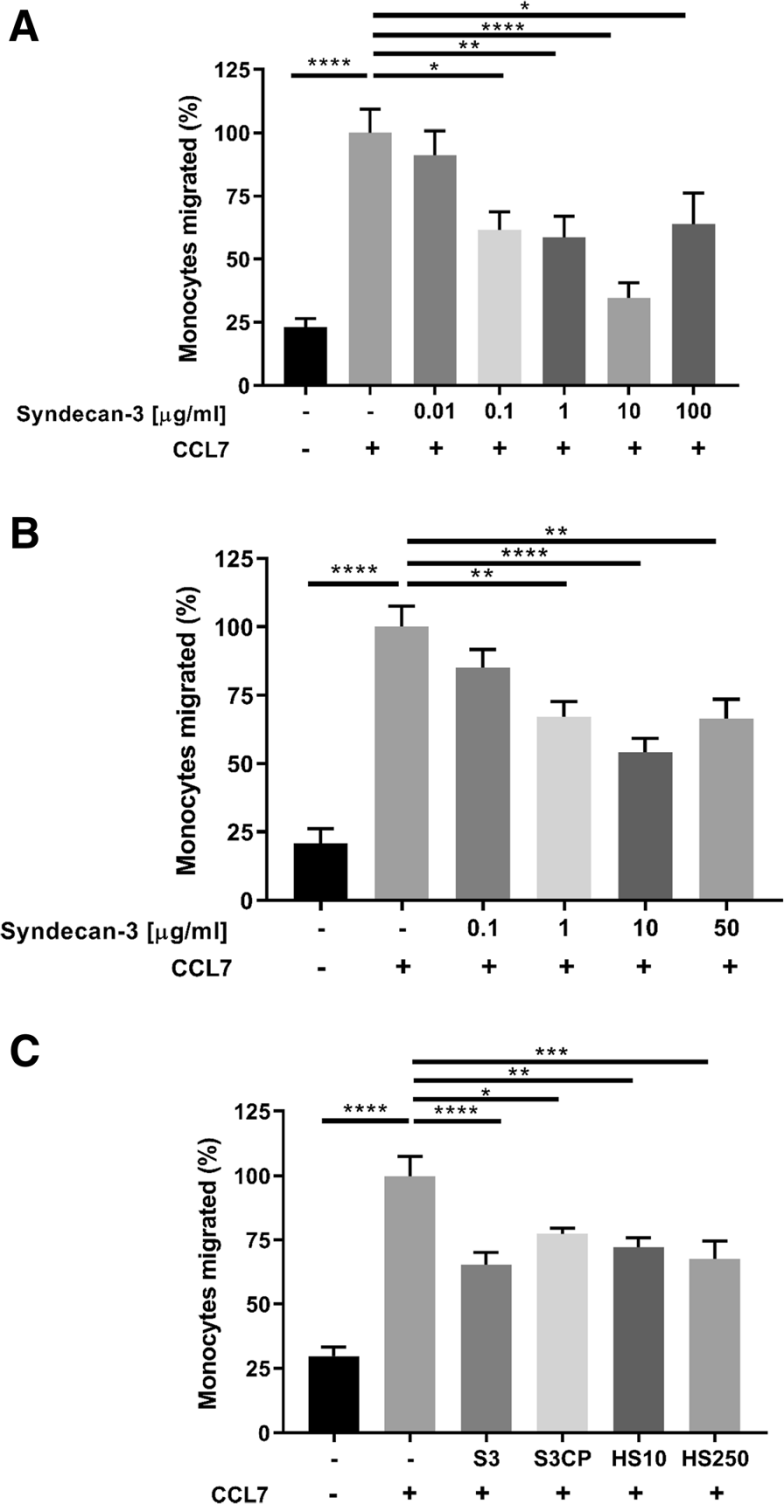
Anti-migratory effects of syndecan-3 in vitro. **a** Migration of THP-1 cells to CCL7 (100 ng/ml) with increasing amounts of syndecan-3 in a Boyden chamber chemotaxis assay. The stars represent a significant ANOVA with Dunnett’s post-test (*P < 0.05, **P < 0.01, ****P < 0.0001). **b** The same assay was carried out using PBMCs and migration across endothelial cell-coated 96-well inserts. The stars represent a significant ANOVA with Dunnett’s post-test (**P < 0.01, ****P < 0.0001). **c** The 96-well format was used to help identify the functional components of syndecan-3 whereby the core protein and HS chains were added individually. Intact syndecan-3 and the syndecan-3 core protein were added at 10 μg/ml. Heparan sulfate was added at 50 and 250 μg/ml as indicated. The stars represent a significant ANOVA with Dunnett’s post-test (*P < 0.05, **P < 0.01, ***P < 0.001, ****P < 0.0001). For **a**–**c**, the columns represent mean values, with standard errors (*n* = 3 separate experiments performed in triplicates). Results are displayed as a percentage of the positive control. S3, syndecan 3; S3CP, syndecan 3 core protein; HS10, heparan sulfate at 10 μg/ml; HS250, heparan sulfate at 250 μg/ml. In all experiments, CCL7 [50 ng/ml] was added to all but the negative control (−). The positive control contained chemokine alone (+)

To characterise the role of shed syndecans in inflammation, soluble syndecans 1–4 were individually added to in vitro migration assays. In the Boyden chamber, the addition of only syndecan-3 to the basal chamber significantly reduced the levels of monocyte (THP-1) migration in response to CCL7 (ANOVA *P* < 0.0001; Fig. 2a). The maximum inhibitory effect was at 10 μg/ml (*P* < 0.0001). At this concentration, there was no significant difference to baseline migration levels. No other member of the syndecan family produced such an effect at any concentration (up to 100 μg/ml); in addition, there were no significant effects of syndecan-1, syndecan-2, syndecan-3 or syndecan-4 on CCL2-stimulated THP-1 cell migration.

The same significant inhibitory effect was observed in the more complex endothelial transmigration assay using PBMCs at a syndecan-3 concentration of 1, 10 and 50 μg/ml (*P* < 0.01, *P* < 0.001, *P* < 0.01 respectively; Fig. 2b). In an attempt to understand the functionality of syndecan-3, the individual components (syndecan-3 core protein and HS chains) were added individually to the basal chamber with CCL7 to observe which had an effect. Figure 2c shows that the individual components did not elicit the same level of response as the intact syndecan-3 (*P* > 0.0001). The condition that mimicked intact syndecan-3 most closely was when HS was added at 250 μg/ml (*P* < 0.001); however, this was calculated to be approximately 5 times the amount as present on the intact syndecan-3. The core protein and HS at 10 μg/ml elicited less significant reductions in migration (*P* < 0.05 and *P* < 0.01 respectively).

### Administration of soluble syndecan-3 reduces disease severity in animal models of RA in vivo

**Fig. 3:**
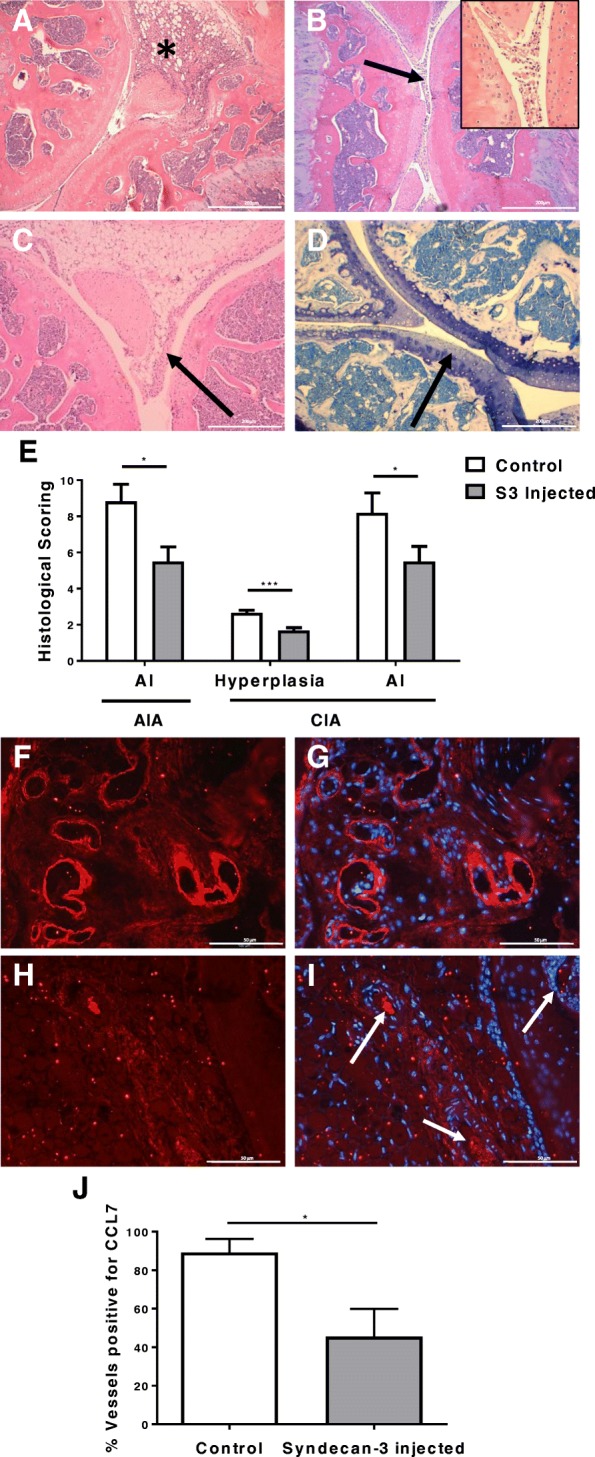
Therapeutic effects of syndecan-3 in the antigen-induced arthritis (AIA) and collagen-induced arthritis (CIA) mouse models. Histological sections were analysed for any effects on inflammation and tissue damage after a syndecan-3 intra-articular injection. Parameters measured for AIA were **a** thickening of the synovium*, **b** exudate in the joint space (arrow) with insert showing higher magnification, **c** hyperplasia of the synovial lining layer (arrow) and **d** loss of proteoglycans from articular cartilage (arrow showing loss of toluidine blue staining). The histological scores for 3 days post-injection are displayed in **e** (means and standard errors are shown, *n* = 4 mice in each group for AIA and *n* = 10 mice for CIA; *P < 0.05; *** P < 0.001; AI, arthritis index). **f**, **g** Blood vessels positive for CCL7 in the control (PBS injected) AIA group. **h**, **i** Blood vessels negative for CCL7 staining in the test (syndecan-3 injected) AIA group, as indicated by arrows. **g**, **i** Merged DAPI images. **j** Five random fields of view per mouse scored based on the percentage of the blood vessels positive for CCL7 staining on synovial endothelial cells after PBS (control) or syndecan-3 intra-articular injection (data are means with standard errors, *n* = 7 AIA mice per group; *P < 0.05). The magnification bars in **a**–**d** represent 200 μm and **f**–**i** 50μm

To identify any effects in biological systems of inflammation, soluble syndecan-3 was administered in two inflammatory mouse models. In the first model (AIA), an intra-articular injection of soluble syndecan-3 significantly reduced overall histological parameters of disease as measured by an arthritis index in comparison with an intra-articular injection of PBS (*P* = 0.04: Fig. 3a–e). To ascertain whether the injected syndecan-3 reduced the expression of CCL7 in the blood vessels, histological sections were stained with anti-CCL7. The joints injected with syndecan-3 contained significantly reduced levels of CCL7 in the blood vessels in comparison with those injected with PBS (*P* = 0.011; Fig. 3f–j). In the second inflammatory model (CIA), multiple intravenous (IV) injections were also sufficient to significantly reduce histological parameters of disease as measured by the arthritis index (*P* = 0.045; Fig. 3e). Individually, the histological scores of intimal hyperplasia were significantly reduced after syndecan-3 injections (*P* = 0.0005; Fig. 3e) whereas other individual scores were reduced but not significantly.

**Fig. 4:**
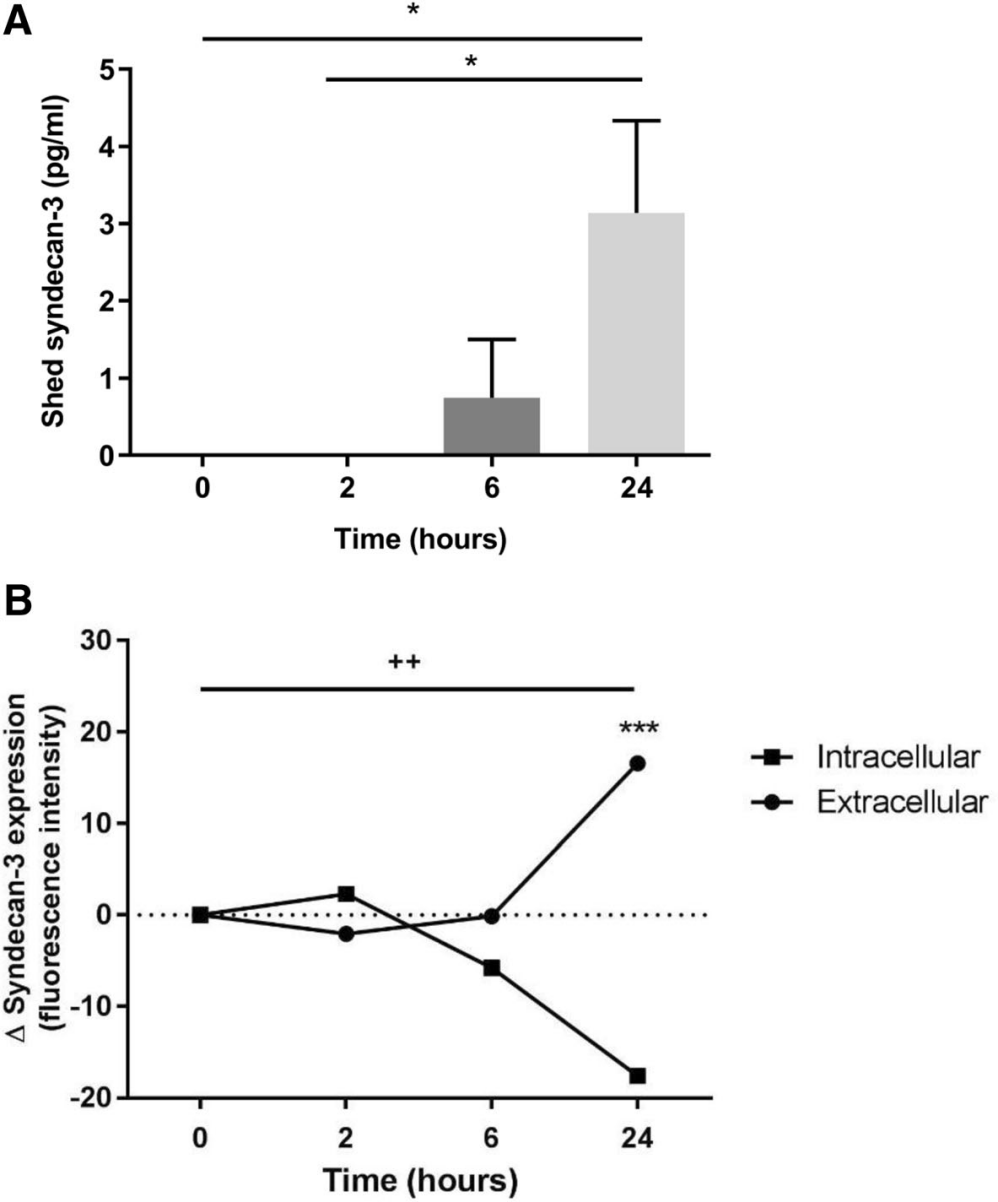
Effects of TNFα on syndecan-3 shedding. **a** Endothelial cells were treated with or without TNFα [100 ng/ml] supplemented media for up to 24 h. The media was then subjected to an ELISA to detect syndecan-3. The graph depicts TNFα-stimulated shedding values minus those of controls in the absence of TNFα at each time point. The bars represent mean values with standard errors (*n* = 3 independent experiments). The stars represent a significant ANOVA followed by Tukey post-test (*P <* 0.05). **b** Again, endothelial cells were cultured with and without TNFα-supplemented media. This time, the level of intracellular and extracellular syndecan-3 was assessed via flow cytometry in comparison with cells treated with control media. Data points represent percentage change of cells positive for syndecan-3 intracellularly or on the extracellular surface (fluorescence intensity; means with standard errors; *n* = 3 independent experiments). The three stars represent a significant two-way ANOVA (without repeated measures) with Tukey post-test (***P < 0.001) depicting a highly significant difference between intracellular and extracellular expression at 24 h. The plus symbol represents significant differences (via one-way ANOVA with Tukey post-test) within the extra- and intracellular expression profiles at 24 h (^++^*P* < 0.01)

### Exposure to TNFα increases endothelial syndecan-3 surface expression and shedding

**Fig. 5:**
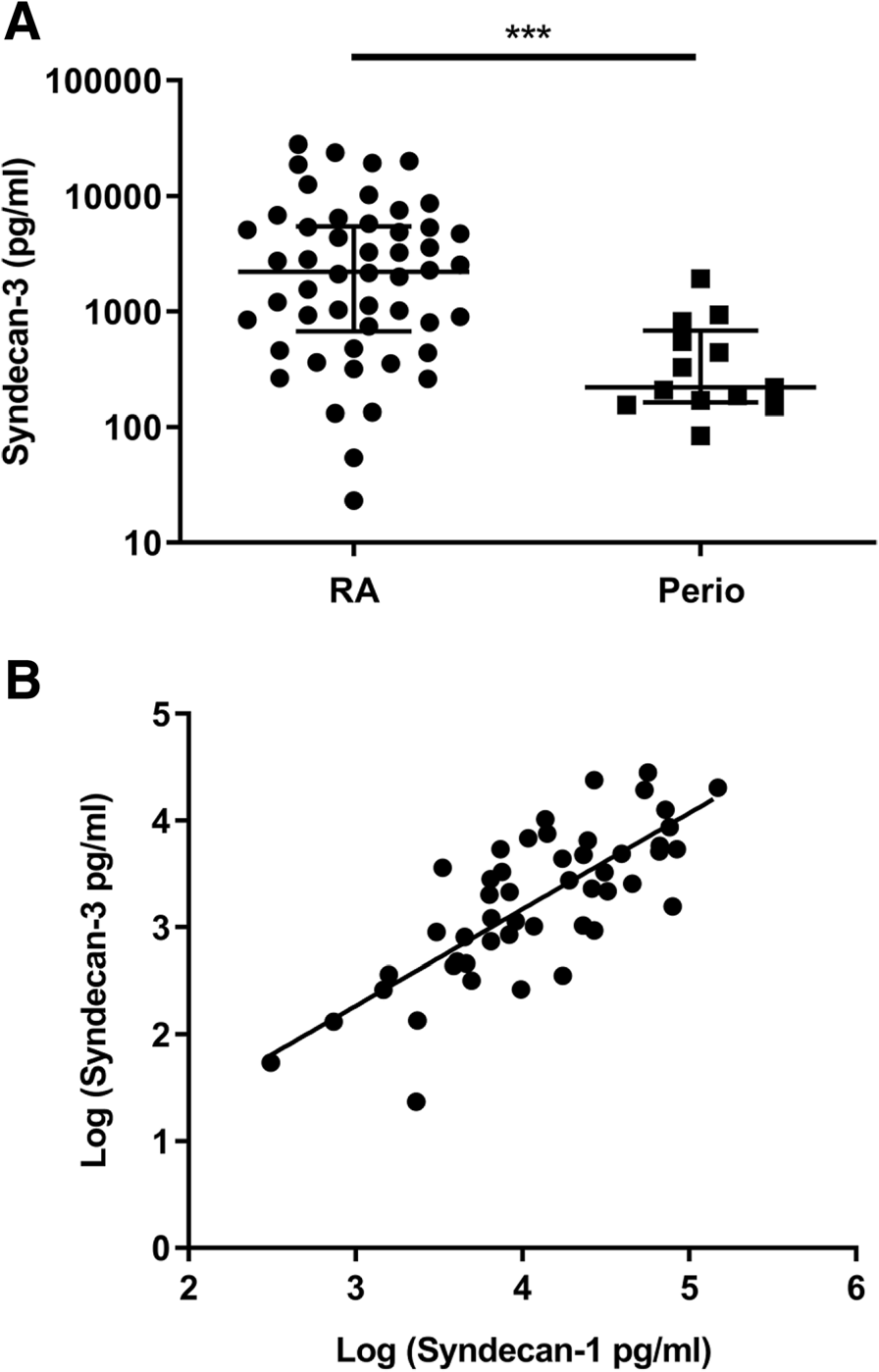
Soluble syndecan-3 occurs in the sera of patients with inflammatory autoimmune diseases. **a** Levels of syndecan-3 in the sera of patients with rheumatoid arthritis (RA; *n* = 47) and periodontitis (Perio; *n* = 13). Data show medians and 95% confidence intervals. ***P < 0.001 (Mann Whitney *U* test). **b** Correlation in the serum levels between syndecan-3 and syndecan-1 in RA sera (correlation coefficient *r* = 0.76; *P* < 0.0001)

To further characterise the role of syndecan-3 in inflammation, hBMECs were stimulated with TNFα for 24 h. The culture medium was analysed for soluble syndecan-3, and cellular syndecan-3 mRNA and protein were also analysed. Over 24 h, the rate of syndecan-3 shedding significantly increased (ANOVA *P* < 0.05; Fig. 4a) in response to TNFα stimulation, being elevated at 6 and 24 h. Over the same time course, the level of intracellular syndecan-3 protein expression reduced by approximately 20% (*P* < 0.01) while the surface expression increased by the same value (*P* < 0.01; Fig. 5b). The difference between intracellular and cell surface expression was maximal at 24 h (*P* < 0.001). The level of mRNA expression in response to TNFα over 24 h did not change (Additional file 1: Table S1).

### Syndecan-3 is shed in human serum

To find out if shed syndecan-3 occurs in vivo in chronic inflammatory disease, sera were collected from patients with RA and periodontitis and analysed by ELISA. Syndecan-3 was detected in both patient groups, and significantly more was present in the sera of RA patients in comparison with those with periodontitis (*P* = 0.004; Fig. 5a). The median syndecan-3 level was 2219 pg/ml in RA and 220 pg/ml in periodontitis. A significant positive linear correlation was observed between the levels of shed syndecan-1 and syndecan-3 in RA patient sera (correlation coefficient *r* = 0.76; *P* < 0.0001; Fig. 5b).

## Discussion

This current study has attempted to further characterise the role that soluble syndecan-3 plays in inflammation as well as explore the possibility of using it as a therapeutic agent. Membrane-bound HSPGs on ECs bind chemokines and participate in leukocyte extravasation by influencing the transport of chemokines across the endothelium and presenting them to the blood leukocytes [8, 10, 12]. Here, we show that soluble syndecan-3 also has the potential to bind to both CC and CXC chemokines involved in the above roles. Our IFT data reveal little difference in the affinity (K_d_) between the chemokines CCL7, CCL2 and CXCL8 and their interaction with syndecan-3. While the chemokine binding to syndecan-3 was similar to HS, some variation was present for CCL2 which showed lower affinity for HS compared to syndecan-3. Chemokine binding to heparin, HS and other GAGs has been well documented [17, 39–42]. However, to our knowledge, this is the first study to characterise the chemokine-binding affinity of a HSPG, which by definition contains GAG chains covalently attached to a core protein, representing how most GAGs occur in the body.

In addition to the roles already attributed to the shedding of syndecans, as stated in the introduction, we proposed an anti-inflammatory mechanism whereby shed syndecan-3 can compete for chemokines limiting their availability to recruit inflammatory cells. In a study by Xu et al. [14], endogenously shed syndecan-1 bound to inflammatory chemokines (CCL7, CCL11, CCL17) attenuating allergic lung inflammation through inhibiting chemokine mediated T cell migration. Combining this finding with a tissue’s potential to induce chemokine-binding sites on specific syndecans led to the idea of testing of their anti-inflammatory potential in vitro and in vivo. From the commercially available syndecans, only syndecan-3 elicited anti-inflammatory effects on monocytic cells and PBMCs in two chemotactic assays in a dose-dependant manner. We propose that soluble syndecan-3 binds to free chemokines and selectively interferes with GPCR-chemokine binding depending on the receptor ligand affinity. This would result in less leukocyte transmigration. Soluble syndecan-3 may therefore be acting as a competitor, trap or decoy molecule in a strategy utilised by the body to further regulate the inflammatory response. Our chemotactic assay concerning individual syndecan-3 components revealed an enhanced effect of the core protein and the HS chains being present together in the HSPG molecule, whereby together, a stronger anti-migratory response was observed than individually. Reasons as to why syndecan-3 was the only syndecan in the family to elicit significant reductions in migration could relate to the length of the HS chains, as those of syndecan-3 are the longest, as calculated from the molecular mass and size from SDS-PAGE (R&D Systems website). Kuschert et al. [17] identified a relationship through solid-phase binding assays whereby chemokine affinity positively correlates with HS chain length. This explanation suggests therefore that the longer syndecan-3 HS chains are more successful in binding chemokines and interfering with the chemokine-GPCR complex. This theory does not however factor in the important intricacies of the GAG chain make-up. This is something that is widely reported as being hugely influential in determining affinities. However, as all our commercially acquired syndecans were synthesised in the same cell type (mouse myeloma cell line, NSO-derived), and that current understanding of chain composition and polymerisation is dependent on the HS enzymes present in the cell’s Golgi bodies at the time of synthesis, one would postulate the GAG composition to be comparable. Syndecan-3 is also known to host chondroitin sulfate chains on the core protein; however, previous reports have highlighted lower chemokine affinities than HS [17, 42].

It needs to be mentioned that endogenously produced syndecans under normal or inflammatory conditions can be modified, for example, in the endothelial cells in RA [29]. The present study uses commercially produced syndecans expressed in mouse myeloma cells, so it is possible that these may contain modifications different from those occurring in vivo.

More recent evidence suggests that chemokine-GAG interactions are more complex than first thought. There are two ways that syndecan-3 may be inhibiting monocyte migration in vitro. Firstly, syndecan-3 may be simply sequestering CCL7. Secondly, CCL7 interaction with syndecan-3 may be interfering with its ability to bind to its high-affinity GPCR receptors [43–45]. Since the ability of HS to bind CCL7 was overall not significantly different from that of intact syndecan-3 (Fig. 1c), their ability to sequester CCL7 was broadly similar. In addition, the presence of the core protein within syndecan-3 enhanced the inhibitory effect on monocyte migration more effectively than HS in vitro (Fig. 2c), suggesting that this HSPG may also be interfering with the ability of CCL7 to bind to its GPCR chemokine receptors on monocytes.

In our two in vivo models of inflammation, the addition of soluble syndecan-3 had therapeutic effects reducing the overall disease severity. This may relate to the reduction in CCL7 staining in the synovial vasculature by syndecan-3, reducing the expression of the chemokine by/on the endothelial cells, which in turn could reduce disease severity. In RA pathology, there is thickening of the synovial intimal layer largely due to increased macrophage numbers [46, 47]. These are recruited as monocytes from the synovial blood vessels and migrate to the intimal layer where they accumulate. The finding that the addition of soluble syndecan-3 reduces intimal layer thickening in CIA may be related to reduced recruitment of monocytes due to this HSPG-binding chemokines like CCL7. However, further experiments showing syndecan-3 binding CCL7, and other mediators, in vivo are warranted to confirm this interaction which was shown in vitro by our IFT and ELISA binding data and migration assays.

It is evident that conflicting reports of the effects of syndecans, soluble or membrane-bound, do exist in the literature, whereby some appear pro-inflammatory while others appear anti-inflammatory. This disparity is likely attributed to the tissues in which syndecans are expressed or type of inflammation. Kehoe et al. report a pro-inflammatory membrane-bound syndecan-3 in the synovium, yet it is anti-inflammatory in the skin and cremaster muscle [16]. Furthermore Li et al. also report a pro-inflammatory function of a shed syndecan-1 binding CXCL8, directing migration and confining neutrophils to the sites of lung injury. It is apparent therefore that syndecans may play various paradoxical roles depending on the tissues in which they are expressed, whether they are membrane-bound or soluble and finally what physiological state the tissues are in. Our study highlights the potential merit of utilising syndecans as a novel therapeutic intervention strategy; however, further work is required to realise the limitations including any potential tissue-specific effects, dose efficacy, half-life and the merits of various administration routes.

In the current study, two routes of syndecan-3 injection were used: IV and intra-articular. CIA is a systemic model of RA involving more than one joint; therefore, it was decided that IV route of injection would be appropriate. Whereas the AIA model affects one joint (the knee); therefore, it was considered more relevant to inject syndecan locally by intra-articular administration. It was of interest to compare these two routes of administration, and both had positive effects on disease outcome.

Research by Patterson et al. has already revealed an induced chemokine-binding site on endothelial syndecan-3 in the RA synovium which was not present on the other syndecans within the tissue. This suggests that ECs are capable of modifying the chemokine-binding affinity of syndecans under different physiological states, most likely through altering the activity of NDST enzymes which has already been documented for other syndecans in different tissues and diseases [48, 49]. Here, we show in vitro that in response to the inflammatory stimulus TNFα, intracellular syndecan-3 stores are transported to the EC surface while, at the same time, increasing levels of syndecan-3 are shed from the EC surface to the surroundings. We hypothesise that this is a potential mechanism to introduce more or replace syndecans on the EC surface with higher affinity HSPGs for chemokines, resulting in higher levels of leukocyte recruitment. In contrast, the shed syndecan-3 could act as an anti-inflammatory effector by binding chemokines and limiting their pro-migratory activity.

Finally, the results showed that shed syndecan-3 does occur in vivo in the human autoimmune/inflammatory conditions of RA and periodontitis. Furthermore, differences appear to exist between inflammatory diseases suggesting the potential of syndecans to be diagnostic markers of diseases. RA levels of shed syndecan-3 are tenfold higher than in periodontitis. Since chemokine levels are elevated in the serum of RA patients, it is possible that syndecan-3 binding is saturated and addition of exogenous syndecan-3 could bind further free chemokines and limit their ability of recruiting leukocytes to inflamed sites. In addition, syndecan-3 is particularly expressed in joint tissues and with multi-joint involvement in RA it might be expected that shed levels to be higher in the serum. Whereas in periodontitis, the disease is more site-specific, being the gingiva that is involved. In periodontitis, the lower levels of syndecan-3 suggest other types of syndecans may be shed, reflecting different expression profiles between inflamed tissues [24, 28]. In addition, there may be positive relationships between syndecans in terms of biomarkers since the levels of syndecan-3 and syndecan-1 correlated in RA sera.

## Conclusions

This study characterises the binding of chemokines to soluble syndecan-3 and shows this soluble HSPG inhibits leukocyte migration in vitro and reduces disease severity in animal models of RA. Therefore, soluble syndecan-3 may have potential therapeutic effects in inflammatory diseases like RA.

## Additional file

Additional file 1: **Table S1.** Effects of TNFα on endothelial cell syndecan-3 RNA gene expression. Endothelial cells were treated with and without TNFα [100 ng/ml] for up to 24 h and RNA subjected to quantitative PCR. An ANOVA with Dunnet post-test did not give any significant differences between time points. (PDF 130 kb)

## Data Availability

The datasets used and/or analysed during the current study are available from the corresponding author on reasonable request.

## Abbreviations

AIA: Antigen-induced arthritis
CIA: Collagen-induced arthritis
EC: Endothelial cell
GAG: Glycosaminoglycan
hBMEC: Human bone marrow endothelial cell
HS: Heparan sulfate
HSPG: Heparan sulfate proteoglycan
IFA: Incomplete Freund’s adjuvant
IFT: Isothermal fluorescence titration
RA: Rheumatoid arthritis
SAB: Standard assay buffer
TEER: Trans-endothelial electrical resistance
TNFα: Tumour necrosis factor α
