# Supplementary material for: Soluble syndecan-3 binds chemokines, reduces leukocyte migration in vitro and ameliorates disease severity in models of rheumatoid arthritis
Source: Arthritis Res Ther. 2019 Jul 12;21:172. doi: 10.1186/s13075-019-1939-2 (PMC6625118; doi:10.1186/s13075-019-1939-2)
Supplement: Supplementary file 1 — Table S1. Effects of TNFα on endothelial cell syndecan-3 RNA gene expression. Endothelial cells were treated with and without TNFα [100 ng/ml] for up to 24 h and RNA subjected to quantitative PCR. An ANOVA with Dunnet post-test did not give any significant differences between time points. (PDF 130 kb) [file 13075_2019_1939_MOESM1_ESM.pdf]

Supplementary Material

Table S1

Supplementary table 1. Effects of TNFα on endothelial cell syndecan-3 RNA gene expression.

Endothelial cells were treated with and without TNFα [100 ng/ml] for up to 24 hours and RNA subjected to quantitative PCR. An ANOVA with Dunnet post-test did not give any significant differences between time points.

Time (hours)	Mean $2^{-\Delta\Delta CT}$	Std Error	Lower 95% CI of mean	Upper 95% CI of mean
0	1	0	1	1
1	0.87	0.05	0.64	1.09
2	1.02	0.10	0.57	1.47
6	0.92	0.09	0.53	1.32
24	0.92	0.14	0.30	1.55
